# 

**Published:** 1845-10

**Authors:** 


### BIBLIOGRAPHICAL RECORD.

1. Report of the Amount of the Causes of Death in Manchester. By John Robertson, Surgeon. 8vo, pp. 23. London, 1845.

2. Letters on the Unhealthy Condition of the Lower Class of Dwellings, especially in large Towns. By the Rev. Charles Girdlestone, A.M. 8vo, pp. 104. London, 1845.

3. The Retrospect of Practical Medicine and Surgery. Vol. XI. Jan. to June, 1845. By William Braithwaite. 12mo, pp. 342. London.

4. On the Nature, Causes, Prevention, and Treatment of Acute Hydrocephalus, or Water Brain Fever. By Thos. Smith, A.M. M.D. Small 8vo, pp. 177. London, 1845.

5. The Cold Water Cure, its use and misuse examined. By Herbert Mayo, M.D. F.R.S. Fc. 8vo, pp. 94. London, 1845.

6. The Chemistry of Vegetable and Animal Physiology. By Dr. G. F. Mulder, translated by Dr. P. F. H. Tromberg, with an Introduction and Notes, by James F. W. Johnstone. London, 1845. Part 2.

7. Surgical and Practical Observations on the Diseases of the Human Foot, with Instruction for their Treatment, to which is added, Advice on the Management of the Hand. By John Eisenberg. 4to. pp. 252. London, 1845.

8. The Half-yearly Abstract of Medical Science, being a Practical and Analytical Digest of the contents of the principal British and Continental Medical Works published in the preceding six Months. Edited by W. H. Ranking, M.D. Vol. 1, Jan. to June. 8vo, pp. 390. London, 1845.

9. Cosmos—a Survey of the General Physical History of the Universe. By Alexander Von Humboldt. Parts 1 & 2. London, 1845.

*A work of the highest scientific merit: we shall notice it at greater length when completed.*

10. The British American Journal of Medical and Physical Science. Edited by A. Hall, M.D. Nos. 1, 2, 3, 4. Montreal, 1845.

11. On Growth or Health and Diseases of Youth. By A. M. Bureaud Riofrey, M.D. 8vo, pp. 163. 1845.

*This little work contains some very useful remarks.*

12. Some Observations on Organic Alterations of the Heart, and particularly on the beneficial Employment of Iron in the treatment of such Cases. By S. Scott Alison, M.D. Fc. 8vo, pp. 62. London, 1845.

13. Lectures on the Theory and Practice of Surgery. By the late Abraham Colles, M.D. Edited by Simon M'Coy. 2 Vols. 12mo. London, 1845.

14. On Diseases of the Liver. By Geo. Budd, M.D. F.R.S. 8vo, pp. 401. London, 1845.

15. A Practical Treatise on Inflammation, Ulceration, and Induration of the Neck of the Uterus, with remarks on the value of Leucorrhœa and Prolapsus Uteri as symptoms of Uterine Disease. By James Henry Bennett, M.D. 8vo, pp. 212. London, 1845.

16. A Treatise on the Diseases and Special Hygiene of Females. By Colombat

DE L'ISERE. Translated from the French by CHARLES D. MEIGS, M.D. 8vo, pp. 719. Philadelphia.

17. On the Nature and Treatment of Gout. By W. H. ROBERTSON, M.D. 8vo, pp. 380. London, 1845.

18. The Naturalist's Library. By Sir WM. JARDINE, Bart. With numerous col. plates, People's Edition, Parts 1 to 5. Highley. London, 1845.

*An exceedingly neat reprint of a most useful and engaging work. It is one of the very best of the "People's Editions:" the excellent letter-press and twelve beautifully coloured engravings for sixteen-pence! The work should be in every good school throughout the kingdom.*

19. A System of Surgery. By J. M. CHELIUS. Translated from the German, with Notes and Observations, by J. F. SOUTH. Parts 3, 4, and 5. London, 1845.

20. The American Journal of the Medical Sciences. Edited by ISAAC HAYS, M.D. July 1845. Philadelphia.

21. History of the British Freshwater Algæ, including descriptions of the Desmideæ and Diatomaceæ, with upwards of one hundred Plates, illustrating the various species. By A. H. HASSALL, F.L.S. 2 vols. 8vo. Highley. London, 1845.

22. The American Journal of Insanity. Edited by the Officers of the New York State Lunatic Asylum, Utica. April and July, 1845.

23. Examination of the Views adopted by Liebig on the Nutrition of Plants. By WM. SELLER, M.D. Edinburgh, 1845.

24. The Medical Examiner and Record of Medical Science. By R. M. HUSTON, M.D. Parts 5, 6, 7, and 8, 1845. Philadelphia.

25. The New York Journal of Medicine and the Collateral Sciences. By C. A. LEE, M.D. July, 1845. New York.

26. Fruits and Farinacea, the proper Food for Man; being an attempt to prove from History, Anatomy, Physiology and Chemistry, that the original, natural, and best Diet for Man is derived from the Vegetable Kingdom. By JOHN SMITH. Small 8vo, pp. 445. London, 1845.

27. Annales de la Chirurgie Française et Etrangere. May, 1845. Paris, Bailliere, 1845.

28. The History of the Middlesex Hospital during the first century of its existence —compiled from the Hospital Records. By ERASMUS WILSON, F.R.S. 8vo, pp. 296. London, 1845.

29. Elements of Chemical Analysis, Qualitative and Quantitative. By EDWARD ANDREW PARNELL. 8vo, pp. 520. 2nd Edition. London, 1845.

30. The Pharmaceutical Latin Grammar. By ARNOLD JAMES COOLEY. 12mo, pp. 132. 1845.

*Calculated to be very useful to medical students.*

31. Medicina Gymnastica. By CHARLES EHRENHOFF. 8vo, pp. 18.

32. Lettre sur la Syphilis. Par F. L. RATIER. 8vo. pp. 68.

33. The Human Female Ovary. By FRANK RENAUD, M.D. 8vo, pp. 20.

34. Forty-ninth Report on the Friend's Retreat, near York. 1845.

35. Fifteenth Report of the Belfast Asylum. 1845.

36. Medical and Physiological Problems. By WM. GRIFFIN, M.D. & DANIEL GRIFFIN, M.D. 8vo, pp. 360. 1845.

37. Dr. Copland's Dictionary of Practical Medicine. Part X. 1845.

38. A View of the Formation, Discipline, and Economy of Armies. By the late ROB. JACKSON, M.D. With a Memoir of his Life and Services. 8vo, pp. 425. 1845.

39. Traité des Angusties ou Retrecissements de l'Uretre, leur traitement rationnel. Par le Dr. LEROY D'ETIOLLS. Paris, 1845.

40. Consultation Medico-legale sur quelques signes de Paralysies vraies, et sur leur valeur relative. Par M. MACLOUGHLIN, D.M. 2me. Edition. Paris, 1845.

41. The Veterinarian. Numbers for July, August, and September.

42. The St. Louis Medical and Surgical Journal. Edited by Drs. LINTON and MC PHEETERS. July, 1845.

43. Tight-lacing and its Consequences. By H. WHITFIELD, M.R.C.S. Pp. 22. 1845.

*May be read with advantage by the public.*

44. Annual Report of the Medical College of Bengal. Session 1844-5.

*This Report gives a very encouraging account of the progress of medical education in Bengal.*

